# Glacier change threatens Central Asia’s water towers

**DOI:** 10.1016/j.isci.2026.114727

**Published:** 2026-01-17

**Authors:** Qifei Zhang, Yaning Chen, Zhi Li, Gonghuan Fang, Yanyun Xiang, Congjian Sun

**Affiliations:** 1 School of Geographical Sciences, Shanxi Normal University, Taiyuan 030031, China; 2 State Key Laboratory of Desert and Oasis Ecology, Xinjiang Institute of Ecology and Geography, Chinese Academy of Sciences, Urumqi 830011, China; 3 Xinjiang Laboratory of Lake Environment and Resources in Arid Zone, College of Geographic Science and Tourism, Xinjiang Normal University, Ürümqi 830054, China; 4 School of Public Administration, Shanxi University of Finance and Economics, Taiyuan 030006, China

**Keywords:** Earth-surface processes, Glacial processes, Glacial landscapes, Hydrology, Remote sensing

## Abstract

Glaciers in the Tien Shan serve as vital “water towers” for Central Asia, but their rapid retreat under climate warming threatens regional water security. This study investigated glacier changes and evaluated their impacts on regional water resources using multi-temporal imagery, integrated with glacier, hydrological, and climatic datasets. Results show widespread and accelerating glacier mass loss, rapid expansion of glacial lakes, and pronounced spatial divergence in runoff responses across basins. The outer mountain ranges experienced particularly strong glacier shrinkage and lake-area growth. Basins with high glacier coverage generally exhibited increasing runoff, whereas less-glacierized basins showed unstable or decreasing runoff, especially where meltwater contributions are approaching peak levels. Overall, these findings demonstrate that glacier retreat compromises the long-term stability and sustainability of Central Asia’s water resources by depleting cryospheric storage, altering hydrological processes, intensifying glacial lake development, and accelerating the shift toward declining water availability after peak melt.

## Highlights

• Glaciers across the TS have experienced widespread and accelerated shrinkage
• Glacial changes strongly influence alpine lakes, river runoff, and water storage
• Glacier change threatens the stability and long-term sustainability of water resources

## Graphical abstract

**Figure:**
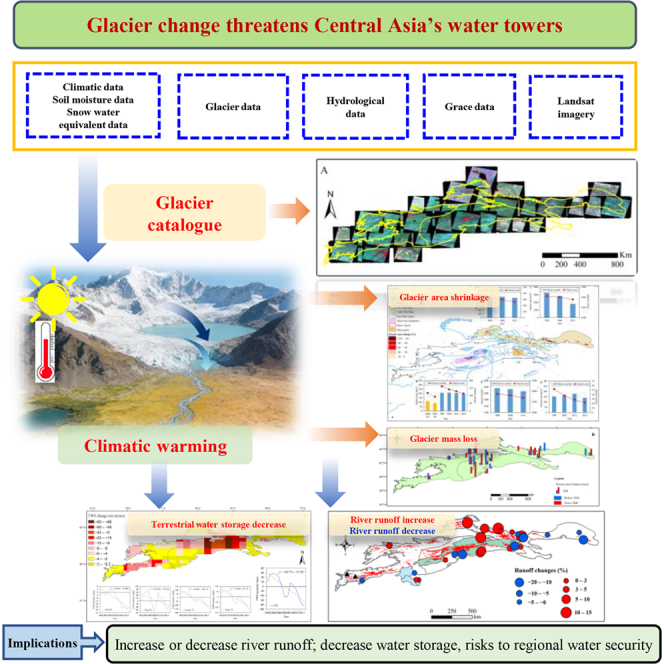

## Introduction

Mountain glaciers are widely recognized as sensitive indicators of climate change^1^ and serve as crucial reservoirs of freshwater^2^^,^^3^^,^^4^ that protect downstream ecosystems and human activities from drought stress.^5^^,^^6^ Over the past century, mountain glaciers worldwide have undergone substantial reductions in both area and mass,^7^^,^^8^^,^^9^ leading to profound impacts on hydrological regimes and water security.^10^^,^^11^

The Tien Shan (TS), with its status as “water tower” of Central Asia, plays a pivotal role in maintaining regional water resources.^12^^,^^13^ Glaciers in this region feed major rivers such as the Amu Darya, Syr Darya, Ili, and Tarim River, providing essential meltwater during dry seasons. However, ongoing climate change has triggered significant glacier and snow cover loss.^2^^,^^3^^,^^14^^,^^15^ Since the mid-19^th^ century, TS glaciers have experienced continuous retreat, with approximately 97.52% of them shrinking due to rising temperatures and shifting precipitation patterns.^16^^,^^17^^,^^18^^,^^19^^,^^20^ Accelerated climate warming has caused extensive glacier retreat,^7^^,^^21^ snow cover reduction, and earlier snowmelt, fundamentally altering the hydrological cycle across the mountain system.^22^^,^^23^ These changes have intensified meltwater fluxes, expanded glacial lakes, and heightened the risk of glacial lake outburst floods,^24^^,^^25^ while simultaneously affecting downstream runoff stability and ecological security.^13^^,^^26^

To assess these impacts, numerous studies have focused on glacier mass balance, runoff contributions, and downstream water availability in the TS.^2^^,^^3^ Conventional approaches include field monitoring, glacier inventories, and hydrological modeling based on climatic relationships.^27^^,^^28^^,^^29^ Recent advances in satellite remote sensing, such as GRACE-based terrestrial water storage (TWS) retrievals and multi-source cryosphere datasets, have further enhanced the ability to quantify regional water balance and its climate-driven dynamics.^20^^,^^30^ However, the vast transboundary extent of the TS exhibits pronounced geographical and climatic heterogeneity. The scarcity of meteorological and hydrological stations, particularly in high-altitude areas, poses significant challenges in effectively assessing the impacts of climate change on water resources in Central Asia. Despite recent advances, several scientific challenges remain. First, the spatial heterogeneity of glacier-fed runoff under different climatic conditions is still poorly quantified. Second, it remains unclear how glacier retreat and associated hydrological shifts influence the long-term dynamics of terrestrial water storage and runoff regimes across Central Asia’s mountain basins. As glacier mass loss accelerates and the coupling between glacier melt, snowmelt, and precipitation becomes increasingly complex under climate warming, uncertainties in attributing water resources changes to specific cryospheric processes continue to grow, underscoring the urgent need for glaciological and hydrological observations.

To address these challenges, this study integrates multi-source satellite observations and hydrological datasets to evaluate the impacts of glacier shrinkage on water resources in the TS. This study aimed to achieve the following objectives: (1) investigate spatiotemporal variations of glacier area and mass to climate change within the global warming context, (2) analyze representative runoff variations across typical catchments, and (3) assess the impact of glacier changes on regional lakes, river runoff, and water resources. By combining these analyses, we aim to attribute the observed water storage changes to glacier retreat and quantify their hydrological implications under ongoing climate warming. The results provide new insights into glacier-runoff linkages in data-scarce mountainous regions and offer a scientific basis for sustainable water resource management and climate adaptation in Central Asia.

### Study area

**Figure 1:**
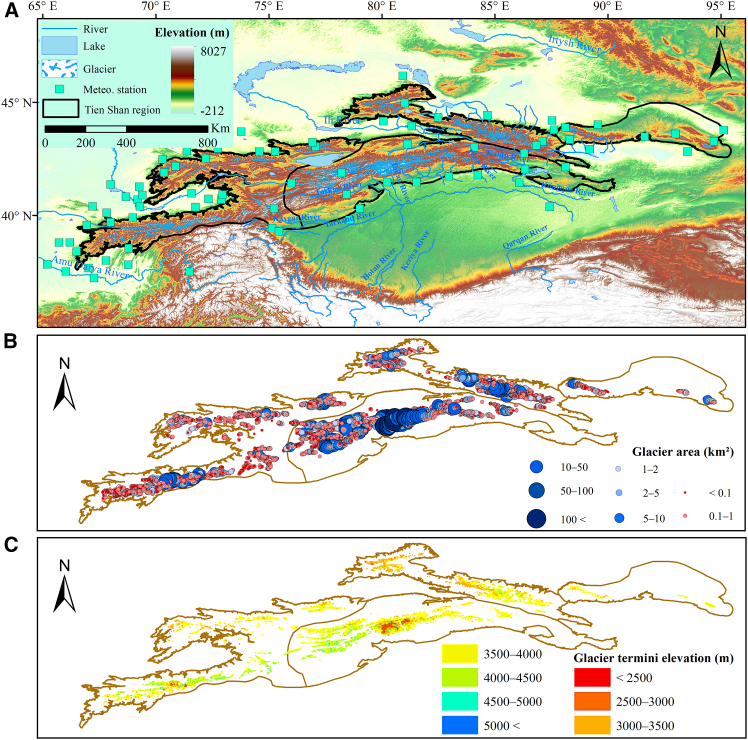
Location of the study area (A) Distribution of glaciers, lakes, rivers, and meteorological stations. (B) Scales of glacier areas in different sub-regions (East, North, Central, West TS). (C) Distribution of glaciers across different elevation zones.

The Tien Shan region, located between 69 and 95°E and 39–46°N in the hinterlands of Eurasia, is one of the world’s major mountain systems. It spans across Xinjiang (China), Kazakhstan, Kyrgyzstan, and Uzbekistan, with a north-south width of 250–350 km and an east-west length of approximately 2,500 km. Based on geographical characteristics, the TS is generally divided into four sub-mountains: East TS, North TS, Central TS, and West TS, together forming the largest mountain-system in Central Asia (Figure 1). The East TS stretches from Hami City to Fukang County, encompassing the Harlik Mountain, Barkol Mountain, and Bogda Mountain. The Bogda Peak (5,099 m), the highest point in this sub-region, rises above elevations ranging from 284 to 5,099 m, where glaciers cover about 0.33% of the region area. The North TS region spans from Turpan’s Yuergou in Xinjiang to the Zhetysu Alatau, featuring elevations of 1,082–5,246 m and a glacier coverage of 2.81%. The Central TS, spanning from the Gangou area to the Fergana Mountains, includes Tomur Peak (7,443 m), the highest region in TS. Elevations range from 966 m to 7,443 m (an average elevation of 2,574 m), and the glaciers cover 4.01% of the area—making it the most glaciated area in TS region, feeding several major rivers, e.g., the Yarkant River and Kaidu River. Central TS is also the most developed and concentrated area of glaciers in the TS, supporting multiple rivers dominated by meltwater from valley glaciers, including the Yarkant River, Kumalak River, Tailan River, Weigan-Kuqa River, Dina River, and Kaidu River Basins. West TS is located west of Central TS, extending from the northeast of Issyk-Kul Lake to the Kashkadarya and Surkhandarya areas of Uzbekistan. The elevation range in this area is 896–5,667 m, and the regional glacier coverage is 1.46%. Overall, the TS is an extensive and topographically complex region with an average elevation of 2,430 m and peaks exceeding 7,000 m, characterized by diverse landscapes of mountains, valleys, and basins.

Known as the “Water Tower of Central Asia,” the TS receives comparatively abundant precipitation in its high-altitude regions under the influence of westerlies,^31^ which sustains extensive glaciers, snowfields, and permafrost. In contrast, intermontane basins and valleys receive much less precipitation, reflecting the strong spatial heterogeneity of the regional climate. Water resources in this region are primarily derived from glaciers and snow melt in high mountains, precipitation in mid-mountain areas, and groundwater recharge from bedrock fissures in lower elevations. These inputs collectively feed numerous rivers across Central Asia and play a vital role in maintaining the regional water cycle, thereby providing freshwater resources for nearly 100 million people. Climatically, the TS is characterized as a temperate continental arid to semi-arid region, with an annual mean temperature of 1.44 °C, mean annual precipitation of 315 mm, hot summers, and extremely cold winters.

## Results

### Spatiotemporal variations of glaciers in Tien Shan

**Figure 2:**
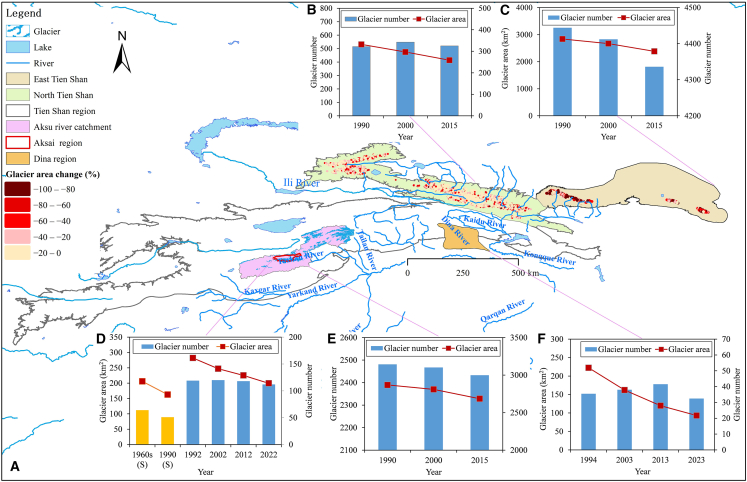
Spatial variations of glaciers across the TS region from the 1960s to 2023 (A) Spatial variations of glacier areas across the TS region. (B and C) Spatial variations of glacier areas in the East and North TS from 1990 to 2015. (D–F) Temporal variations of glacier number and area in the Central TS (D) Naryn River Basin (the orange bar represents the consistent glacier number in 1990 and the 1960s according to the Soviet Union Catalog, while the blue bar indicates the total glacier number for 1990, 2002, 2012, and 2022); (E) Aksu River Catchment (1990–2016); and (F) Dina River Basin (1994–2023).

Since 1990, the glaciers in the TS have shown a significant retreat trend (Figure 2). From 1990 to 2015, the total glacier area in East TS ranged from 332.04 to 258.86 km^2^, with a reduction of 73.18 km^2^ (22.04%). The total number of glaciers increased from 515 in 1990 to 520 in 2015, while glacier mass decreased from 1.53 × 10^13^ kg in 1990 to 1.17 × 10^13^ kg in 2015, representing a shrinkage rate of −0.94% yr^−1^. During different periods, the glacier area in East TS decreased by approximately 10.59% between 1990 and 2000, but the decrease expanded to 12.80% from 2000 to 2015 (Figure 2C).

Over the past 25 years, both the total number and area of glaciers in North TS have exhibited notable negative trends (Figure 2B). The total glacier area decreased from 2,838.94 km^2^ in 1990 to 2,382.51 km^2^ in 2015, representing a reduction of 456.43 km^2^ (16.08%). The total number of glaciers declined from 4,444 to 4,336, while glacier volume decreased from 1.44 × 10^14^ kg to 1.20 × 10^14^ kg, with a reduction rate of 0.66% yr^−1^. Notably, the glacier retreat accelerated from 2000 to 2015 compared with 1990–2000, with the glacier area decreasing by 6.02% from 1990 to 2000, and then increasing to 10.70% from 2000 to 2015.

The glaciers in the outer ranges of the central Tien Shan region have experienced more pronounced retreat. From 1990 to 2016, glaciers in the Aksu River Catchment experienced substantial retreat (Figure 2C). The glacial area declined from 2,868.67 km^2^ in 1990 to 2,685.14 km^2^ in 2016, representing a reduction of 6.40% over the past 26 years, with a decrease rate of 0.25% yr^−1^. Glacier volume decreased from 2.38 × 10^14^ kg to 2.21 × 10^14^ kg, representing a reduction of approximately 0.17 × 10^14^ kg during the same period. From the 1960s to 2022, the glaciers in the Naryn River Basin of Central TS experienced a significant retreat (Figure 2C). Based on glacier dynamics derived from the Soviet Glacier Catalog of the 1960s and corresponding 1990 data, the number and area of glaciers decreased by 13 and 43.15 km^2^, respectively. Since 1990, the number of glaciers in this basin has declined from 119 in 1990 to 112 by 2022, while the glacier area decreased from 282.08 km^2^ to 199.52 km^2^. Over the past 30 years (1990–2022), the total glacier area diminished by 82.55 km^2^, with a speed of −0.91% yr^−1^. From period from 1994 to 2023, the glaciers in the Dina River Basin of Central TS (with a mean glacier area of 0.16 km^2^) experienced a significant retreat of −1.04% yr^−1^.

In addition to the shrinkage of glacier area, glacier termini in the TS have migrated to higher elevations. In the East TS, the average elevation of glacier termini rose from 3,720 m in 1990 to 3,771 m in 2000 and further to 3,794 m in 2015, presenting an increase of approximately 74 m. Similarly, the elevation of glacier termini in the North TS also showed a continuous upward trend. The average elevation of glacier termini across the entire mountain range increased from 3,656 m in 1990 to 3,664 m in 2000, and then to 3,693 m in 2015, showing a rise of nearly 37 m.

### Spatiotemporal variations of glacier mass balance in Tien Shan

**Figure 3:**
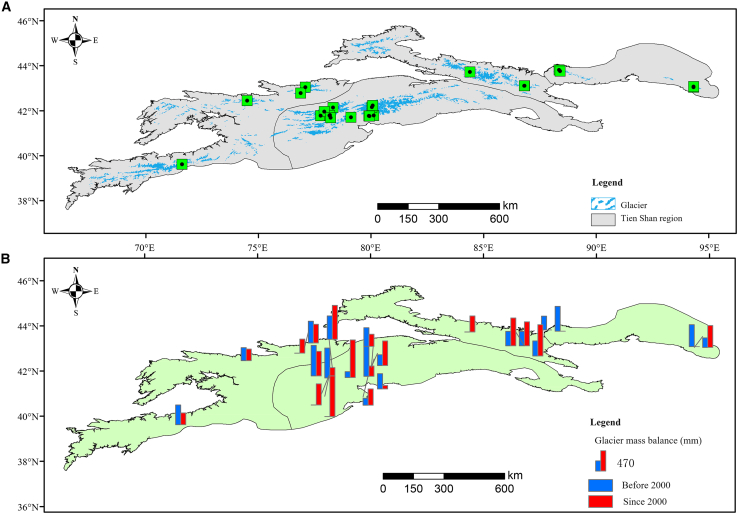
Distribution and variations of glacier mass balance across the TS region between 1990–2000 and 2000–2015 (A) Distribution of glacier mass balance sites. (B) Variation of glacier mass balance between 1990–2000 and 2000–2015. Spatial patterns and rates of glacier mass balance change before and after the year 2000, highlighting regional heterogeneity with more negative balances in eastern and central regions. Data are presented in mm water equivalent per year (mm w.e. yr^−1^).

Glacier mass balance in the TS has exhibited a persistent decline over recent decades. However, the rate of mass loss shows pronounced spatial variability: the eastern and central mountainous regions experienced a much greater decrease compared with the western mountains (Figure 3). Prior to 2000, the average annual glacier mass balance in the East TS region was −405 mm w.e. yr^−1^. Specifically, the glaciers in the Bogda Peak area had a multi-year average mass balance of −445 mm w.e. yr^−1^, showing a significantly higher value than that of the Harlik Mountains in the East TS, which exhibited a multi-year average of −365 mm w.e. yr^−1^. In North TS, negative glacier mass balance intensified markedly after 2000, increasing from −337.66 mm yr^−1^ before 2000 to −642.18 mm w.e. yr^−1^ since 2000, nearly doubling the average annual loss. In contrast, all monitored glaciers in West TS have shown a smaller decrease in mass balance since 2000, with a multi-year average of −323.44 mm w.e. yr^−1^, compared with −419 mm w.e. yr^−1^ before 2000.

In Central TS, the glacier mass balance showed a marked decrease from 1990 to 2000, with a multi-year average of −447 mm w.e. yr^−1^, but the rate of mass loss significantly slowed after 2000, dropping to −218.33 mm w.e. yr^−1^, particularly evident in the Tomur Peak area. However, the presence of surge type glaciers in the central and western TS caused substantial spatial and temporal variability in glacier ablation rates before and after 2000. For instance, over the past 50 years, 39 glaciers in the Tomur Peak area, the upper reaches of the Toxkon River, the AK-SHIRAK region, and the northern part of Issyk-Kul Lake in TS have experienced surges, most of which were concentrated around Tomur Peak.^36^ The largest Southern Inylchek Glacier in the Tomur Peak exhibited a mass loss of approximately 0.43 ± 0.10 m w.e. yr^−1^ from 1975 to 1999. However, since 1990, this speed slowed, reaching 0.28 ± 0.46 m w.e. yr^−1^ during 1999–2007. In contrast, the adjacent Northern Inylchek Glacier, which had a balance of 0.25 ± 0.10 m w.e. yr^−1^ from 1975 to 1999, experienced accelerated mass loss from 1999 to 2007, up to 0.57 ± 0.46 m w.e. yr^−1^,^34^ mainly due to a surge event between October 12 and November 13, 1996.

This was confirmed by multi-temporal Landsat images, showing that the glacier terminus advanced by approximately 3.7 km during the surge. After the surge, due to the rapid decrease in glacier terminus elevation, the glacier area exposed below the mass balance line increased, accelerating ablation and mass loss in that the portion of the glacier. This explains why the mass loss of the Northern Inylchek Glacier has significantly accelerated since 2000, while that of the Southern Inylchek Glacier has experienced a slowdown trend. Since 2000, the upstream areas of the Kumalak and Tailan Rivers, as well as the entire Tomur Peak region, have generally shown a slowdown in glacier mass loss.^37^

From 1959 to 2019, both Urumqi Glacier No. 1 in the North TS and Tuyuksuyskiy Glacier in the West TS exhibited negative mass balances. Over the past 60 years, the average annual mass balance change rate for Urumqi Glacier No. 1 was −13.61 mm w.e. yr^−1^, significantly higher loss than the −5.12 m w.e. yr^−1^ observed for Tuyuksuyskiy Glacier. The multi-year average annual mass balances for Urumqi Glacier No. 1 and Tuyuksuyskiy Glacier were −422.97 mm w.e. and −335.15 mm w.e. yr^−1^, respectively. Cumulative mass balance changes indicated that Tuyuksuyskiy Glacier thinned by approximately 25.62 m from 1959 to 2019, while Urumqi Glacier No. 1 thinned by approximately 20.44 m during the same period.

### Spatiotemporal variations of terrestrial water storage, lakes, and river runoffs in Tien Shan

**Figure 4:**
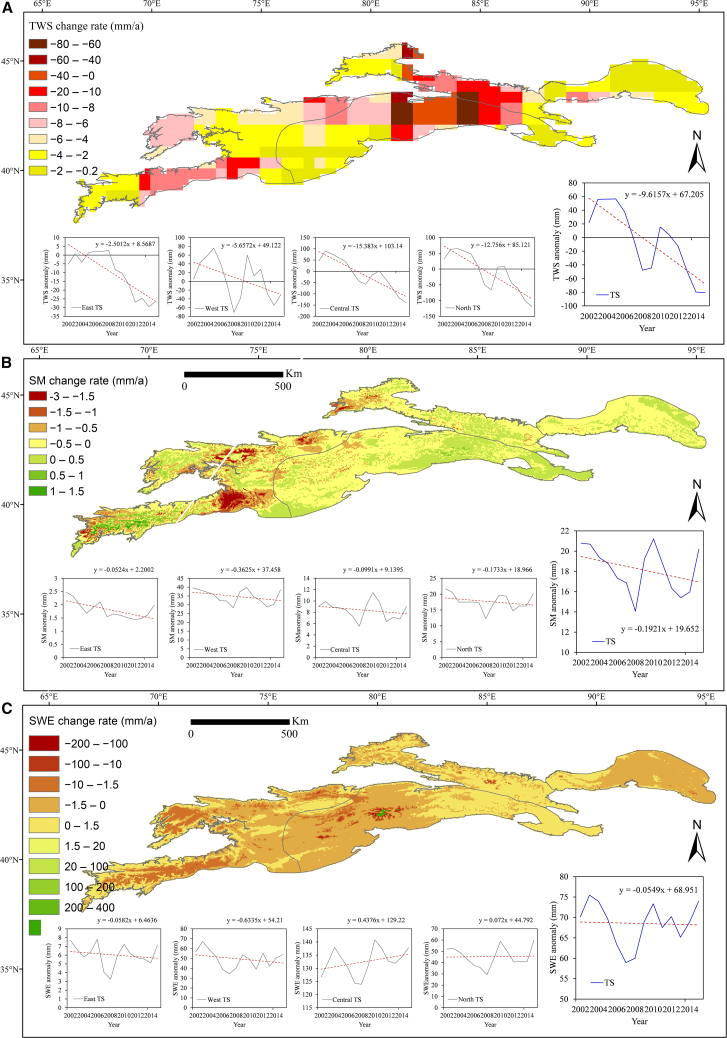
Variations of terrestrial water storage (TWS) across the TS region from 2002 to 2015 (A) Spatial variations of TWS derived from GRACE data. (B) Spatial variations of the soil moisture (SM). (C) Spatial variations of snow water equivalent (SWE). The pronounced decline in TWS, in contrast to the relatively minor changes in SM and SWE, indicates that glacier mass loss is the dominant contributor. Trends were evaluated using the Mann-Kendall test and Sen’s slope estimator.

Analysis of GRACE data reveals a persistent decline in terrestrial water storage (TWS) anomalies across the TS since 2002, averaging −0.87 mm per month (Figure 4). Distinct spatial heterogeneity in TWS variations is evident across the mountain range. In most parts of the TS, the rate of water storage decline is less than 10 mm yr^−1^, whereas the central region exhibits a pronounced decrease, reaching up to −80 mm yr^−1^.

**Figure 5:**
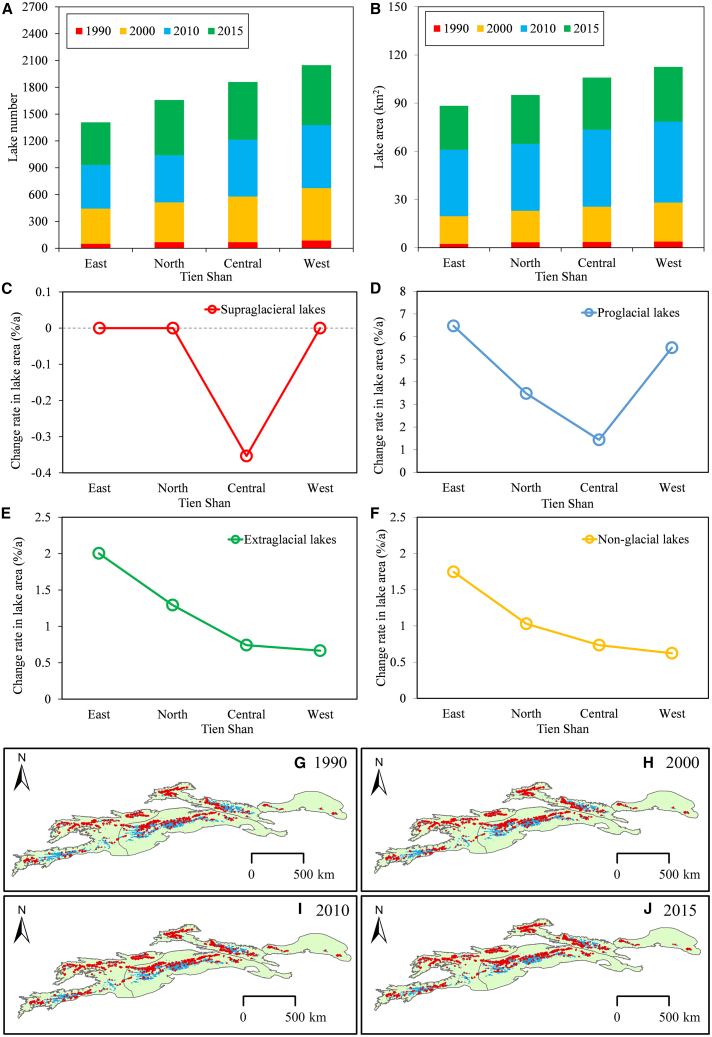
Changes in alpine lake number and the area of alpine lakes in the TS region from 1990 to 2015 (A and B) Variations in the number and area of glacier lakes. (C–F) Annual change rates in supraglacial, proglacial, extraglacial, and non-glacial lake areas, respectively. (G, H, I, and J) Spatial distribution of alpine lakes during the years 1990, 2000, 2010, and 2015, respectively.

Since 1990, both the total number and area of glacial lakes in the TS have exhibited positive trends (Figure 5; Table S1). The number of lakes increased by 712 (41.66%), and the area expanded by 26.73 km^2^ (25.95%), with annual increase rates of 1.67% and 1.04%, respectively. A comparison of glacial lake areas across different regions from 1990 to 2015 revealed significant disparities in both the number and area of lakes among various TS regions. Central TS saw a notable increase, with the number of lakes rising by 217 and the area expanding by nearly 10.27 km^2^. In the North and West TS, the total number of glacial lakes increased by 192 (7.09 km^2^) and 194 (6.70 km^2^), respectively. In contrast, East TS experienced the smallest growth, with increases of just 39 lakes and 1.44 km^2^. Regarding the rates of expansion during different periods, East and North TS exhibited the highest growth rates for glacial lakes, at 2.44% and 1.65% yr^−1^, respectively. The West TS followed with a rate of 0.98% yr^−1^, while Central TS had the slowest expansion rate at 0.87% yr^−1^.

From 1990 to 2015, glacial lakes (including supraglacial, extraglacial, and proglacial) exhibited significant increases in both number and area, expanding by 45.45% and 27.08%, respectively. In contrast, non-glacial lakes showed modest growth, with the number increasing by 23.92% and their area expanding by approximately 19.01%. Among the different types of glacial lakes, proglacial lakes recorded the highest rates of increase, with annual growth rates of 4.40% yr^−1^ in number and 2.41% yr^−1^ in area. Extraglacial lakes followed, expanding at rates of 1.48% yr^−1^ for number and 0.84% yr^−1^ in area. Non-glacial lakes had the slowest growth rates, increasing at 0.96% yr^−1^ in number and 0.76% yr^−1^ in area during the same period. Notably, supraglacial lakes showed a decline, with reductions of 0.08% yr^−1^ in number and 0.15% yr^−1^ in area.

When comparing the changes in alpine lakes across different regions of the TS from 1990 to 2015 (Figure 5), both periglacial and proglacial lakes exhibited significant expansion trends, outpacing the growth rates of non-glacial lakes. Particularly, proglacial lakes have shown notable expansion, with annual rates of 6.47%, 5.51%, 3.49%, and 1.44% in the East, West, North, and Central TS, respectively, over 25 years. In comparison, the annual expansion rates of extraglacial lakes (2%) and non-glacial lakes (1.75%) in East TS were relatively higher than those in other regions (Figures 5D and 5E). Notably, no supraglacial lakes were found in the eastern and northern TS, while those in Central TS displayed a declining trend, shrinking at a rate of 0.35% yr^−1^. The spatial correspondence between TWS decline and glacial lake expansion highlights the role of glacier melt in modulating regional water storage dynamics.

**Figure 6:**
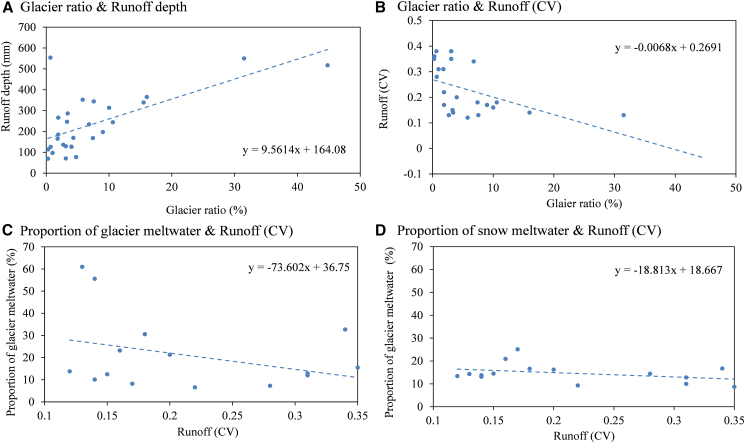
Characteristics of river runoff in the TS region based on varying proportions of glacier area and snow meltwater from 1990 to 2015 (A) Relationship between glacier coverage (%) and mean annual runoff depth (mm). (B) Relationship between glacier coverage (%) and the coefficient of variation (CV) of annual runoff. (C) Relationship between the proportion of glacier meltwater in total runoff (%) and runoff CV. (D) Relationship between the proportion of snowmelt water in total runoff (%) and runoff CV.

**Figure 7:**
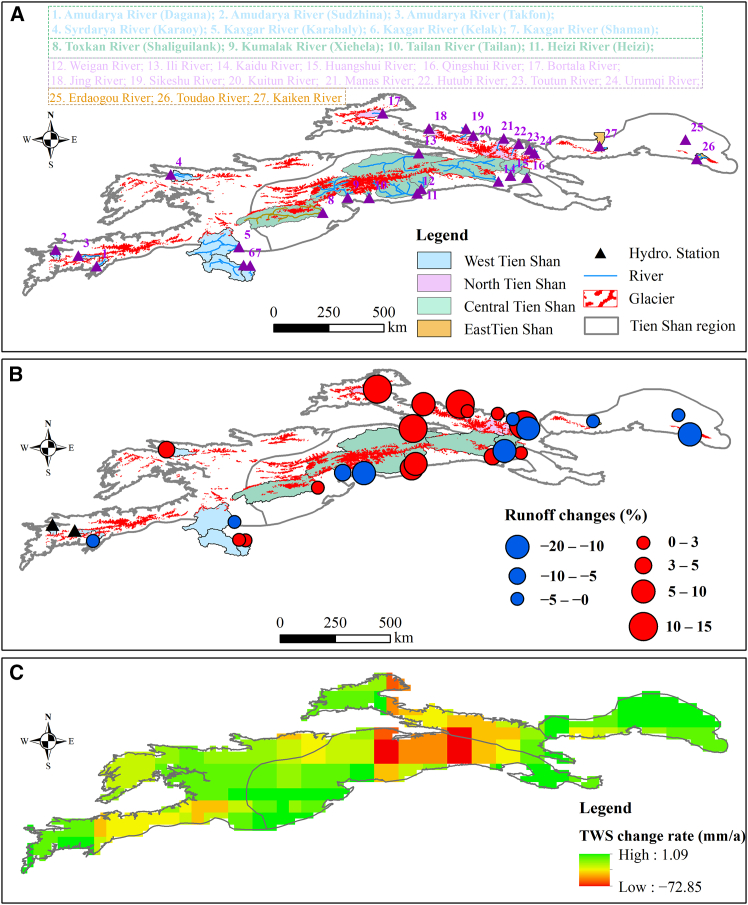
Distribution of typical rivers and variations of river runoffs and TWS in the Tien Shan between the periods 1990–2000 and 2000–2015 (A) Distribution of major rivers, hydrological stations, and glaciers across the Tien Shan region. (B) Variations in river runoff between the periods 2000–2015 and 1990–2000. (C) Spatial variations of total water storage between 2003 and 2015.

River runoff analyses further illustrate the hydrological impact of glaciers. Basins with a higher proportion of glaciers in the TS exhibited lower CV (coefficient of variation) values, indicating stronger river runoff stability (Figure 6). In comparison to rivers in other regions of the TS, those in the East TS showed some instability, with most CV values exceeding 0.3. Spatially, as shown in Figure 7A, the Yushugou River Basin, which has the highest glacier coverage area (7.42%), had a runoff CV value of 0.18, demonstrating relative stability. In contrast, the Kaiken River Basin (with a glacier proportion of 0.65%) and the Toudaogou River Basin (with a glacier proportion of 3.1%) had the highest CV values, reaching 0.38. The Baiyang River Basin, with a glacier coverage proportion of 6.8%, experienced relatively large interannual variations in annual runoff (with a CV value as high as 0.34), primarily due to precipitation variability in the watershed (with CV values ranging from 0.23 to 0.4), where the maximum annual runoff over the years is about three times the minimum runoff.

## Discussion

### Glacier shrinkage exacerbates the loss of water resources

Changes in water resources in the TS are governed by multiple factors, including temperature fluctuations (which influence glacier and snowmelt rates), precipitation changes, and the combined effects of evapotranspiration, glacier meltwater input, and mountainous topography. Among these, glacier meltwater represents a crucial source for glacial lakes and plays an essential role in maintaining their hydrological balance. In the TS, approximately 91% of lakes are located within glacierized catchments. As dynamic components of the cryosphere, glaciers respond sensitively to climate variability, with their accumulation and ablation processes largely regulated by climatic conditions.

Changes in temperature, precipitation, and their temporal patterns have exerted diverse impacts on glaciers, snow cover, and the generation of meltwater and snowmelt runoff in the TS. The hydrological cycles of rivers and lakes primarily sustained by snow and glacier meltwater are expected to intensify, heightening the uncertainty of water resources in Central Asia and amplifying the frequency and magnitude of extreme hydrological events. Under a warming climate, glaciers across High Asia have been retreating at an accelerated rate.^38^ Meanwhile, the number and area of glacial lakes have increased markedly, and their expansion has partially offset regional water resource losses associated with glacier retreat.

TWS variations derived from GRACE represent the integrated effects of multiple hydrological components, including glacier mass changes, snow accumulation and melt, precipitation, evapotranspiration, and SM. However, evapotranspiration in mountainous regions generally exerts a relatively minor influence on total TWS. To assess whether non-glacial factors significantly affected TWS trends, we incorporated additional datasets of SM and SWE. The results indicate that both variables exhibited small interannual fluctuations (SM: −0.19 mm yr^−1^; SWE: −0.05 mm yr^−1^) compared with the pronounced and spatially consistent TWS decline. Moreover, their temporal variations were weakly correlated with GRACE-derived anomalies, indicating that their contributions to total TWS changes were limited. Therefore, although TWS dynamics in the TS are modulated by multiple hydrological processes, the strong spatial coherence between glacier retreat and TWS reduction, together with the minor variability in SM and SWE, confirms that glacier mass loss is the dominant driver of the observed decrease. This finding supports the study’s primary objective of quantifying the impact of glacier retreat on terrestrial water storage in Central Asia’s mountainous regions.

Glaciers constitute a critical component of the cryospheric water reserves in the TS, and their ongoing retreat under climate warming poses a significant threat to regional water availability. Spatially, the rate of TWS decline closely corresponds to the magnitude of glacier loss. Among the subregions, Central TS experienced the largest glacier area reduction (approximately 805.08 km^2^ from 1990 to 2015, accounting for 46.27% of total glacier loss), accompanied by the highest TWS decline rate of −1.36 mm per month. A similar pattern was observed in North TS, where glacier retreat reached ∼456.43 km^2^, and TWS anomalies decreased at −1.14 mm month^−1^. In contrast, West TS underwent moderate glacier loss (∼374.16 km^2^) and a lower TWS decline rate of 5.94 mm yr^−1^. Although East TS contained a smaller glacierized area (73.19 km^2^), higher temperatures accelerated glacier retreat, resulting in a 25.88% reduction in glacier area over 25 years and a TWS anomaly decline of −0.22 mm month^−1^. The smaller glaciers in East TS are particularly sensitive to climatic warming, leading to the most pronounced relative glacier retreat in the region.

Regarding interannual variation in runoff in the TS region, basins with a higher proportion of glacier coverage generally exhibited more stable flow regimes. In the TS, glacier meltwater exerts a stronger influence on runoff stability than snowmelt (Figures 6C and 6D). This is evident in the long-term runoff variations of the Yushugou River in the East TS, the Sikeshu River and Manas River in North TS, and the Kumalak and Tailan Rivers in Central TS. Deng et al.^32^ reported that in the TS region, the Aksu River—with higher glacier coverage—showed a runoff increase of 0.4 × 10^8^ m^3^ yr^−1^ from 1960 to 2010, exceeding that of the Kaidu River (0.17 × 10^8^ m^3^ yr^−1^) and the Urumqi River (0.04 × 10^8^ m^3^ yr^−1^). This illustrates that among the different river runoff components in the TS region, rivers with a high proportion of glacier meltwater have a more pronounced regulatory effect on runoff than rivers with a high proportion of snowmelt recharge. In this study, rivers with high glacier coverage in North, Central, and West TS—where glaciers constitute a substantial portion of the catchment—generally maintained an upward trend in runoff (Figure 7).

River runoff in the TS region heavily relies on glaciers and snowmelt from upstream mountainous areas. Recent climate change has substantially affected glaciers and snow cover, increasing uncertainty in downstream glacial water resources and altering the relative contributions of different sources to total runoff. Under the warming climate, rivers with extensive glacier coverage and a high proportion of glacial meltwater recharge have exhibited a sustained increase in downstream runoff over extended periods. Using climate model projections, Yang et al.^39^ analyzed future runoff variations in 25 basins with varying glacier coverages across the TS and found that runoff trends in highly and moderately glacierized basins are largely controlled by the magnitude of regional warming.

**Figure 8:**
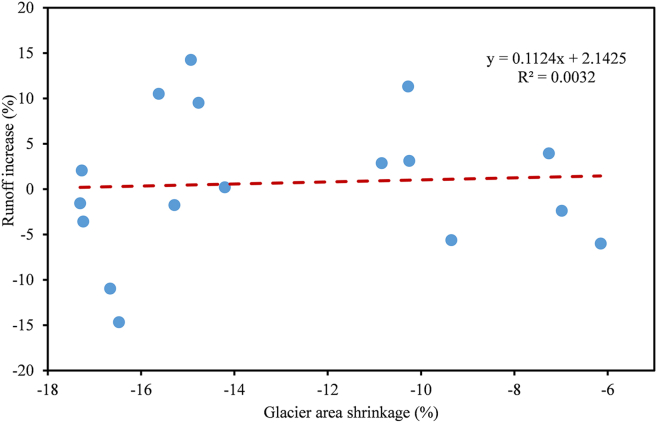
Relationship between the variations of glacier area and river runoffs from 1990 to 2015 Scatterplot illustrates the relationship between the rate of glacier area change (% yr^−1^) and the corresponding percentage change in river runoff across multiple basins.

Against the backdrop of climate warming, nearly all rivers in the North TS have exhibited an upward trend in runoff since 2000, with more pronounced increases corresponding to intensified glacier shrinkage (Figures 7 and 8). For instance, compared with the average annual river runoff from 1990 to 2000, the Bortala, Jinghe, Sikeshu, Manas, and Toutun Rivers increased by 10.03%, 9.19%, 11.57%, 3.00%, and 10.53%, respectively. The Kuitun River experienced the smallest increase, with average annual runoff rising by only 1.48% since 2000, largely due to its relatively low glacier coverage (1.9%) within the watershed. In contrast, the annual runoff of the Urumqi River decreased by 14.26% over the same period. Glaciers in the Urumqi River basin have experienced significant retreat under warming conditions, with the area of Urumqi Glacier No. 1 shrinking by approximately 13.37% between 1990 and 2015. At present, perennial glacier coverage in the river’s source region is 3.4%, contributing only 10.1% to its annual runoff. The substantial reduction in glacier area has markedly diminished the meltwater supply, resulting in decreased runoff in the Urumqi River.

In basins with limited glacier coverage, the contribution of glacial meltwater to river runoff is constrained by the low glacier area in the basin. Consequently, changes in glacier water resources have a limited impact on runoff, and the effect of rising temperatures on streamflow is relatively minor. For example, many rivers in the upstream regions of East TS have low glacier coverage. Although the total glacier area in East TS has retreated significantly (−0.88% yr^−1^) in recent decades, these changes still exert only a limited impact on downstream river runoffs. Previous studies have confirmed that basins with small glacier coverage in East TS have experienced minimal recent changes in runoff.^16^^,^^21^ Kaldybayev et al.^21^ found that in the Karatal River basin of the North TS, where glacier coverage exceeds 5%, runoff exhibits an overall increasing trend, whereas basins with glacier coverage below 2% show declining runoff. Chen et al.^40^ reported significant runoff reductions in the Toudao and Erdao River basins in the Eastern TS, with declines of approximately 12.5% and 6.6%, respectively, since 1998, due to rapid glacier area loss (glacier coverage <1%). Ragettli et al.^41^ found that runoff in the Juncal catchment in Chile will decrease sharply as glaciers reach the ablation peak; however, in the climatically similar Tarfala catchment, where glacier coverage is nearly 30%, flood frequency remains high. Between 1979 and 2002, the runoff of the Kumalak River, with a glacier coverage of 16%, exhibited significantly higher runoff than the Toshkan River, where glaciers covered less than 4% of the basin. Future projections suggest that, due to the relatively high glacier coverage, meltwater from the Kumalak River basin will reach the ablation peak only around the 2050s.^42^

Since 2000, the average annual runoff of rivers with relatively low glacier coverage has generally decreased. For example, the Kaiken River (0.65% glacier coverage in the basin) and the Erdaogou and Toudaogou Rivers (3.1% glacier coverage) have experienced declines of 1.61%, 3.57%, and 16.37%, respectively, relative to the period 1990–2000. However, not all rivers dominated by large-scale glacial meltwater exhibit increasing runoff trends under climate warming. In the Tomur Peak region, the Tailan River (61% glacier coverage in the basin) and the Kumalak River (55.6% glacier coverage in the basin) have seen reductions in average annual runoff of 13.86% and 6.89%, respectively, since 2000 compared with 1990–2000. Collectively, the observed TWS decline, glacial lake expansion, and runoff variability indicate a coupled hydrological response to glacier retreat in the TS. Regions experiencing intense glacier loss display both accelerated lake growth and altered runoff regimes, highlighting the critical role of glaciers in sustaining regional water resources. Yang et al.^39^ analyzed future runoff variations in 25 basins with different glacier coverages across the TS using climate model projections and found that, under climate change, runoff in low-glacier-coverage basins is expected to increase due to higher precipitation. By the end of this century (2071–2100), in most basins with low to moderate glacier coverage, the contribution of glacier melt to total runoff is projected to become negligible (<5%), posing challenges to the stability of regional water supply.

### Glacier variations in response to climate change

Glacier retreat and associated hydrological changes in the TS are primarily driven by regional climatic changes, including rising summer temperatures and shifts in precipitation patterns. Higher air temperatures enhance glacier surface melting and extend the ablation season, while reductions in solid precipitation reduce accumulation, accelerating glacier mass loss. Between 1990 and 2015, the TS experienced a warming rate of 0.30 °C per decade, exceeding the global average of 0.12 °C per decade (IPCC, 2013),^43^ and comparable to the arid regions of northwest China (0.31 °C per decade)^13^ and Xinjiang (0.30 °C per decade).^26^ This amplified warming directly intensified cryospheric degradation and altered regional hydrological processes.

The spatial heterogeneity of glacier retreat reflects regional climatic contrasts. Western TS glaciers are largely influenced by Atlantic westerlies and receive higher winter precipitation, whereas Eastern TS glaciers experience continental arid conditions with limited snowfall and greater temperature sensitivity. Consequently, glacier-fed runoff in eastern basins exhibits earlier and more pronounced seasonal peaks, while western basins maintain relatively stable flow regimes. Accordingly, glacier retreat rates are substantially higher in the East and North TS (0.88% yr^−1^ and 0.64% yr^−1^, respectively) than in the West and Central TS, where larger and higher-altitude glaciers retreat more slowly. Since the late 1990s, strengthened westerly circulation and persistent regional warming have further accelerated glacier thinning and meltwater contributions, particularly in the central and eastern TS.^17^

**Figure 9:**
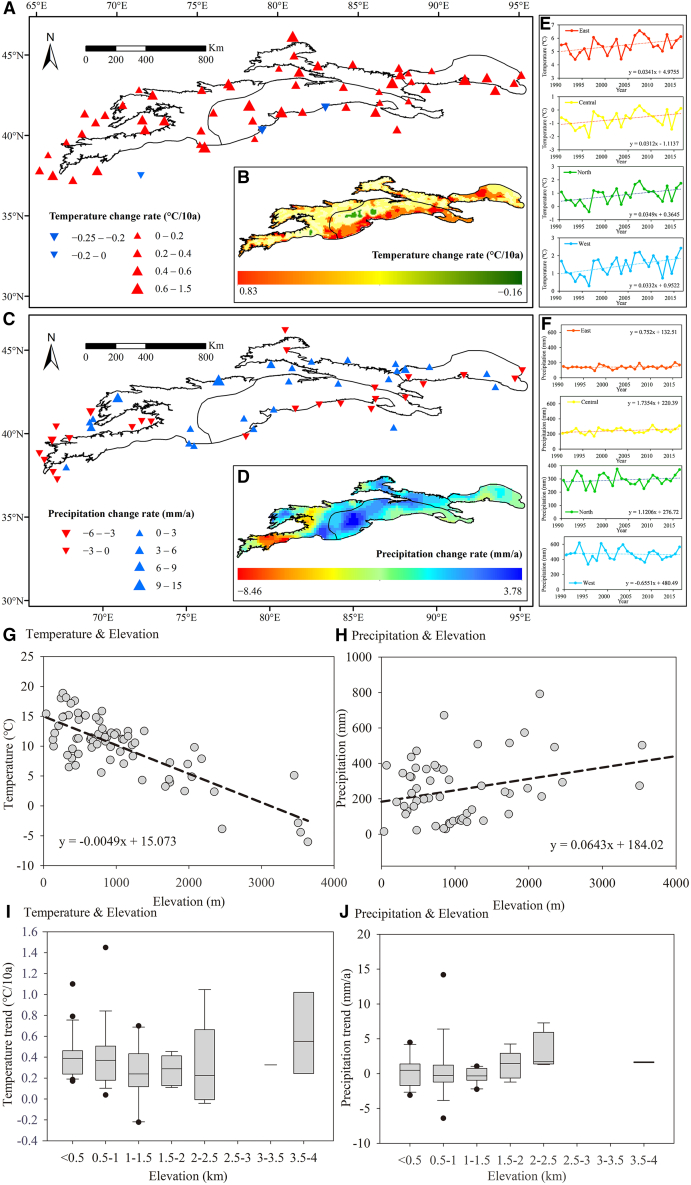
Changes in annual temperature and precipitation in the TS region from 1990 to 2015 (A and B) Spatial trends of annual temperature change rate (°C per decade) from the meteorological station and the ERA5-Interim dataset. (C and D) Spatial trends of annual precipitation change rate (mm per year) from the meteorological station and the GPCC dataset. (E and F) Temporal variations in temperature and precipitation for the East, Central, North, and West TS regions. (G and H) Variations in annual average temperature and precipitation with different altitudes. (I and J) Trends in temperature and precipitation at different altitudes.

Analysis of climatic data from 1990 to 2015 reveals complex spatial trends (Figure 9). Nearly all stations (95%) recorded significant warming, except for the West TS and three low-altitude stations in the South TS. The most pronounced warming occurred in the East TS (0.34 °C per decade), while North and South TS also exhibited rates above the regional average, excluding the East TS. Annual average precipitation increased by ∼16.44 mm, corresponding to a growth rate of 0.69 mm per decade, but with significant spatial variability. Central and southern regions experienced the largest increases, whereas southern outer regions and southwestern western TS showed decreasing trends. Higher temperatures combined with reduced precipitation intensified aridity in outer regions, while inner regions remained humid due to increased glacier melt, snowmelt, and precipitation. These spatial climate patterns drive heterogeneous glacier ablation and mountain lake replenishment.

Altitude strongly influences temperature and precipitation. In high-altitude TS regions, lower temperatures and abundant precipitation support extensive glacier and snow development. Temperature decreases by ∼0.49 °C per 100 m increase in altitude, whereas precipitation rises by 6.43 mm per 100 m (Figure 9M). Warming rates decrease below 2,500 m but accelerate above this altitude (Figure 9P), promoting snow and ice loss and increasing downstream runoff. Precipitation also rises with altitude but exhibits spatial variability (Figure 9Q).

Climate changes affect both glacier accumulation and ablation processes and significantly alters hydrological regimes, including runoff dynamics and water resources. Shifts in precipitation patterns further modulate these processes, although their precise impacts on runoff remain uncertain. Despite a global warming slowdown, the TS has experienced persistent, high-amplitude temperature fluctuations over the past decade, exacerbating ablation of glaciers and snow cover, and reducing snowfall rates. From 1960 to 1998, snowfall rates increased at 0.6% per decade, but since 2000, the snowfall-to-precipitation ratio has decreased by −0.7% per decade.^44^ In mountainous basins, runoff formation is closely linked to these climatic shifts. Under continued warming, changes in precipitation patterns, seasonal distribution, and spatiotemporal dynamics of glacier snow cover are expected to affect river recharge processes, seasonal runoff distribution, and overall water availability.

Overall, sustained glacier change poses a growing threat to the stability of the Central Asia’s Water Tower. Long-term glacier mass reduces the cryosphere’s buffering capacity, and shifts in meltwater contributions are accelerating the transition from increasing to declining runoff, marking the onset of peak water. Concurrently, rapid glacial lake expansion amplifies hydrological processes, while sustained losses in terrestrial water storage reflect a progressive weakening of regional water security. Collectively, these processes indicate that continued glacier recession will increasingly compromise the sustainability and reliability of water resources for downstream ecosystems and human societies.

## Resource availability

### Lead contact

Requests for further information and resources should be directed to and will be fulfilled by the lead contact, Yaning Chen (chenyn@ms.xjb.ac.cn).

### Materials availability

All the materials of this study are available from the lead contact without restriction upon request.

### Data and code availability

• The datasets analyzed in this study are publicly available at https://zenodo.org/records/18035651.
• The original code supporting this study is available from the lead contact upon reasonable request.
• Any additional information required to reanalyze the data reported in this article is available from the lead contact upon reasonable request.

## Acknowledgments

We sincerely thank Daniel E. Lawson for his valuable suggestions regarding glacier extraction methods across the Tien Shan region. We would also like to thank Editage (www.editage.cn) for English language editing. This research was supported by the State Key Laboratory of Desert and Oasis Ecology, 10.13039/501100009958Xinjiang Institute of Ecology and Geography, Chinese Academy of Sciences (G2023-02-01), the 10.13039/501100001809National Natural Science Foundation of China (W2412135, 42130512), and the Major Science and Technology Program of Xinjiang Uygur Autonomous Region (2024A03006-1).

## Author contributions

Conceptualization, Q.Z. and Y.C.; methodology, Q.Z.; investigation, Z.L. and G.F.; writing – original draft, Q.Z.; writing – review and editing, Q.Z. and Y.X.; funding acquisition, Y.C.; supervision, Y.X. and C.S.

## Declaration of interests

The authors declare no competing interests.

## STAR★Methods

### Key resources table

**Table:** 

REAGENT or RESOURCE	SOURCE	IDENTIFIER
**Deposited data**		

Landsat	United States Geological Survey (USGS)/Geospatial Data Cloud site of the Computer Network Information Center	http://www.usgs.gov; http://www.gscloud.cn
Glacier data	Soviet Glacier Inventory/Randolph Glacier Inventory 6.0	http://www.glims.org/RGI/randolph60.html
Glacier mass data	World Glacier Monitoring Service (WGMS); TS Glacier Observation and Experiment Station	https://wgms.ch/contact_wgms/
ERA-Interim dataset (temperature)	European Center for Medium-Range Weather Forecasts	https://apps.ecmwf.int/datasets/
GPCC data (precipitation)	Global Precipitation Climatology Center	https://www.dwd.de/EN/ourservices/GPCC/GPCC.html
Terraclimate data (soil moisture, snow water equivalent)	GEE platform	https://www.nature.com/articles/sdata2017191
Stream data	Regional Hydrological Bureau of China; Global runoff data center	Regional Hydrological Bureau of China; https://www.bafg.de/GRDC/EN/Home/homepage_node.html
Meteorological data (temperature, precipitation)	China Meteorological Data Service Center (CMDC); National Snow and Ice Data Center (NSIDC); National Centers for Environmental Information (NCEI)	http://data.cma.cn/en; ftp://sidads.colorado.edu/pub/DATASETS/NOAA/G02174/; https://www.ncei.noaa.gov/
GRACE	National Aeronautics and Space Administration (NASA)	https://www2.csr.utexas.edu/grace/
Topography	United States Geological Survey (USGS)	https://glovis.usgs.gov/
Geospatial Data	Open-source datasets	https://doi.org/10.5281/zenodo.18035651

**Software and algorithms**		

MATLAB R2025a	MATLAB R2025a	https://www.mathworks.com
ArcGIS 10.8.2	Esri	https://www.esri.com
Python 3.12.3	Python Software Foundation	https://www.python.org

### Method details

#### Satellite data

Please describe here all statistical analysis and software used. We ask authors to indicate in this section where all of the statistical details of experiments can be found (e.g., in the figure legends, figures, results, etc.), including the statistical tests used, exact value of n, what n represents (e.g., number of animals, number of cells, etc.), definition of center, and dispersion and precision measures (e.g., mean, median, SD, SEM, confidence intervals). Also, please summarize in this section how significance was defined, the statistical methods used to determine strategies for randomization and/or stratification, sample size estimation, and inclusion and exclusion of any data or subjects, as well as any methods used to determine whether the data met assumptions of the statistical approach.

Changes in glacier areas and glacial lakes were determined using multitemporal satellite images, primarily from the Landsat imagery. Fifty-seven high-quality Landsat TM/ETM+/OLI scenes (PATH: 137–155; ROW: 28–34) were obtained from the United States Geological Survey (USGS, http://www.usgs.gov) and the Geospatial Data Cloud site of the Computer Network Information Center, Chinese Academy of Sciences (http://www.gscloud.cn). All images were carefully selected to ensure cloud-free conditions during the ablation season, when seasonal snow cover was minimal, thereby minimizing uncertainties in glacier boundary extraction.

To minimize the impact of seasonal snow and cloud cover on the delineation of glacier and mountain lake boundaries, Landsat imagery acquired from July to October was selected, accounting for approximately 95% of the dataset. Although several scenes contained partial cloud cover, they still provided useful information over unobscured areas and were therefore retained for analysis. Missing or low-quality images were replaced with temporally adjacent scenes of comparable quality. This strategy ensured a more complete and consistent extraction of glacier and glacial lake information across the study period.

Additionally, a variety of high-resolution imagery was employed to assist in glacier and lake identification, including WorldView-2 (∼0.5 m resolution), Google Earth, Bing Maps, GeoEye, SPOT-5, and QuickBird (∼1.65 to 2.62 m resolution). These datasets, combined with Landsat imagery, provided detailed topographic and geomorphic information for detecting glacial changes from 1990 to 2015. Additional references included Google and Baidu maps, further aided in identifying glaciers, glacier types, moraines, mountain terrains, lake basins, rivers, clouds, and anthropogenic features (e.g., hydropower stations, reservoirs, ponds, and canals). High-resolution ASTER 1T imagery, sourced from the USGS EarthExplorer, was also used to delineate glacier boundaries in the TS region. The image interpretation was further supported by field investigations and relevant literature.

This study utilized monthly gravity field solutions from the Center for Space Research (CSR) GRACE Release 06 (RL06) mission, with a spatial resolution of 0.25° and covering the period from April 2002 to February 2016. The GRACE twin satellites measure minute variations in Earth’s gravity field, which are primarily caused by mass redistributions of terrestrial water storage, thus providing valuable insights into the global water cycle. The CSR RL06 data were processed using standard procedures, which included replacing the degree-1 and C20 coefficients with more reliable estimates, applying a Gaussian filter for spatial smoothing, and using a P4M6 decorrelation filter to reduce longitudinal stripe noise. Missing monthly data were interpolated using the multi-year mean of the corresponding months,^32^ a method validated for maintaining the continuity of trend analysis in large-scale hydrological studies.

#### SRTM DEM

The Shuttle Radar Topography Mission (SRTM1) Arc-Second Global Digital Elevation Model (DEM) data were used to construct the topographic base for the glaciated basins. These data, provided by the USGS (https://earthexplorer.usgs.gov/), have a spatial resolution of 30 m. The DEM served as the foundation for extracting and quantitatively analyzing changes in individual glaciers.

#### Climatic and stream data

The analysis of climatic changes in and around the TS region relied on annual, monthly, and daily temperature and precipitation records from 33 meteorological stations (Figure 1). Annual and monthly climate data for the Chinese region were downloaded from China Meteorological Data Service Center (CMDC, http://data.cma.cn/en), and the Regional Hydrological Bureau from 1960 to 2015. Climatic data outside China were downloaded from the National Snow and Ice Data Center (NSIDC, ftp://sidads.colorado.edu/pub/DATASETS/NOAA/G02174/) and the National Centers for Environmental Information (https://www.ncei.noaa.gov/). Given the sparsity of meteorological observation stations in the TS, especially in high-altitude areas (> 3,000 m), monthly precipitation data from the Global Precipitation Climatology Center (GPCC, V.2018, https://www.dwd.de/EN/ourservices/GPCC/GPCC.html) from 1990 to 2015 were used. These data helped clarify the spatial patterns of climate variation. The GPCC dataset provides a global precipitation analysis during 1891–2016 based on quality-controlled data from stations in the Global Climate Change Center database, using up to 79,000 stations worldwide. This product is optimized for spatial coverage and has been widely applied in water balance studies. Compared with other datasets,^15^ it offers higher precision with a spatial resolution of 0.25° and performs robustly in complex mountains.^31^ Additionally, monthly gridded surface temperature maps (2 m above ground) were obtained from the ERA-Interim dataset provided by the European Center for Medium-Range Weather Forecasts (ECMMWF, https://apps.ecmwf.int/datasets/). This dataset provides temperature data at a 0.25° horizontal resolution for 1979–2015, calculated from average daily mean temperatures. Soil moisture and snow water equivalent data for the TS region were obtained from the TerraClimate dataset via Google Earth Engine (GEE) platform. The dataset provides globally consistent monthly climate and water-balance variables at ∼4 km resolution.

Monthly surface runoff data spanning 1960–2015 were obtained from various river basins originating in the TS region, including the Aksu (Kumalak and Toxkan Rivers), Kaidu, Huangshui Gou, Weigan, Ili, Boertala, Kuitun, Manasi, Hutubi, Urumqi, Kaiken, and Hami Rivers. The stream data were provided by the Regional Hydrological Bureau of China. Additionally, annual runoff data for rivers outside China, such as the Syr Darya, Amu Darya, and Issky-Kol Rivers, were obtained from the global runoff data centre (https://www.bafg.de/GRDC/EN/Home/homepage_node.html). Notably, the collapse of the Soviet Union in the early 1990s led to the discontinuation of numerous hydrological monitoring stations and routine surveillance programs, significantly affecting the availability and continuity of hydrological data in the region.

#### Glacier data

Glacier boundaries extracted for the 1990–2016 period were verified using the Randolph Glacier Inventory (RGI 6.0) – a global glacier outline compilation developed under the Global Land Ice Measurements from Space initiative (GLIMS). Historical glacier extent for the Naryn River Basin (Central TS) in the 1960s was obtained from the Soviet Glacier Inventory. Representative glacier mass data across the TS (1959–2016) were sourced from World Glacier Monitoring Service (WGMS) publications: Fluctuations of Glaciers and Glacier Mass Balance Bulletin. Data for Urumqi Glacier No. 1 were further supplemented by annual reports from the TS Glacier Observation and Experiment Station (Chinese Academy of Sciences).

#### Methodology

##### Extraction glacier and lake information

Several glacier mapping techniques were evaluated, including band ratio thresholding, supervised and unsupervised classification, normalized difference snow index (NDSI), decision tree analysis, and object-based image interpretation. However, the presence of snow, shadows, moraines, and water often reduces classification accuracy. Therefore, this study employed the band ratio thresholding method, which provides an efficient and reliable means of distinguishing glaciers from clouds and shadows. Subsequently, multispectral images were visually inspected using optimized band combinations to enhance the contrast between glacier surfaces (snow, ice, and debris cover) and surrounding non-glacier areas.

Glacier outlines were delineated on Landsat images from 1990, 2000, and 2015. We initially employed a semi-automated approach using the band ratios to classify glacier and non-glacier areas, which are widely recognized for mapping debris-free glaciers and are commonly used in global glacier inventories.^14^ However, this method is unsuitable for debris-covered glaciers. In such cases, visual interpretation was applied using features such as terminal moraines, meltwater stream heads, glacial lakes, and lateral moraines. Misclassified features (e.g., snow patches, cast shadows, and water bodies) were manually corrected using multispectral band combinations (TM/ETM+ bands 3, 5 and 7; OLI bands 4, 6 and 7) on Landsat imagery.

Uncertainties in glacier mapping primarily arise from the presence of snow, shadows, clouds, water bodies, and debris cover. To minimize these effects, we selected Landsat images with minimal cloud contamination. Since water bodies did not refreeze during the ablation periods, this factor was excluded from further consideration. Misclassified features such as lakes, shadows, and seasonal snow were manually corrected. Debris-covered glaciers are mainly associated with larger valley glaciers and account for approximately 5% of the total glacierized area in the Tomor region. Specifically, the TM3/TM5 (Landsat TM/ETM+) or OLI4/OLI6 (Landsat 8) band ratio was applied to delineate clean ice, while low-reflectance zones near glacier termini were verified using high-resolution Google Earth imagery and RGI inventories to identify debris-covered areas. Multi-temporal images (±1–3 years) were used to confirm glacier boundaries in areas with unclear delineation. All outlines were independently reviewed by two operators to ensure consistency, and discrepancies were reconciled through repeated manual verification, thereby reducing potential subjectivity.

Two significant factors that disrupt the inventory process are snow cover and ice lakes, particularly when mapping moraine-covered regions. Water features were distinguished using slope data from the SRTM1 DEM. Glaciers in the Naryn River Basin during the periods 1990, 2002, 2012, and 2022 were extracted using the Normalized Difference Snow Index (NDSI) method, with further refinement through visual inspection. Although the TS region experiences minimal cloud cover, glacier identification remains challenging, especially in high mountain areas. Therefore, visual inspection was further adopted to refine glacier outlines using high-resolution Google and WorldView-2 imagery. Alpine lakes across the TS region were extracted using the Normalized Difference Water Index (NDWI), and the boundaries and types of glacial lakes were manually refined and classified (Figure S1).

Lake outlines of alpine lakes in the TS were delineated from Landsat images during 1990–2015 based on the Normalized Difference Water Index (NDWI). To ensure the reliability of lake extraction results, high-resolution datasets (including Google Earth imagery and WorldView-2 imagery with ∼0.5 m resolution) and 30 m resolution DEM data were integrated to verify lake boundaries; human-impacted water bodies (e.g., reservoirs and ditches) were excluded to avoid interfering with the analysis of glacial lake change drivers. Detailed methods for calculating lake area changes, estimating extraction uncertainties (e.g., buffer zone method for edge error assessment, Gaussian distribution-based boundary error analysis), and defining lake classification criteria are described in the supplemental information (Figure S1, Methods S1).

##### Analysis of glacier mass balance, terrestrial water storage, and runoff dynamics

To evaluate the impacts of glacier changes on regional water resources in the TS, we integrated multi-source datasets with published glacier mass balance results to estimate glacier volume variations. Glacier mass balance estimates were primarily derived from the Randolph Glacier Inventory, combined with regional observations and previously published datasets.^15^^,^^24^ The calculation process and data sources used for glacier volume and mass balance estimation are provided in the supplemental information (Methods S1).

In assessing regional terrestrial water storage (TWS), we comprehensively accounted for glacier volume changes derived from the above data. TWS variations were obtained from GRACE mascon datasets (CSR RL06). To better isolate the influence of glacier retreat on water storage, we removed the effects of soil moisture (from GLDAS Noah) and snow water equivalent (SWE) (from ERA5-Land) from the total GRACE-derived TWS anomalies. This correction ensured that the residual TWS changes primarily reflected cryospheric mass loss associated with glacier retreat. Temporal trends and spatial heterogeneity of these components were further evaluated using the Mann–Kendall test and Sen’s slope method.

To assess the hydrological impacts of glacier retreat, we examined river runoff variations using long-term hydrological observations and reanalysis data across basins with differing degrees of glacierization. Runoff variability was quantified using the coefficient of variation (CV), where lower CV values indicate greater flow stability. By comparing basins with high glacier coverage (e.g., Tailan River) and low glacier coverage (e.g., Kaiken River), we assessed how glacier meltwater influences interannual runoff dynamics and hydrological stability. The results confirmed that basins with higher glacier coverage exhibit more stable runoff regimes, indicating the buffering role of glaciers in sustaining regional water resources.

All preprocessing procedures—including spatial resampling, temporal smoothing, and uncertainty assessments—are described in detail in the supplemental information.

##### Error estimation

Uncertainties in glacial area measurements were classified as either technical or methodological errors. Technical errors are negligible when satellite images have undergone accurate orthorectification, which applies to Landsat scenes provided by the United States Geological Survey (USGS). Considering that the Landsat Thematic Mapper (TM), Enhanced Thematic Mapper Plus (ETM+), and Operational Land Imager (OLI) imagery underwent standard topographic correction (Level 1T), this study conducted a comprehensive glacier analysis across the entire region rather than a pixel-by-pixel analysis. Consequently, we contend that total registration errors do not substantially affect glacial area measurements. Methodological errors in glacier extraction from remote sensing datasets primarily depend on the spatial resolution and registration accuracy of the imagery. To address gaps caused by poor-quality imagery, multitemporal scenes spanning 1–3 years outside the classification year were used to delineate glacier margins in areas with missing coverage. Finally, all debris-covered glacier outlines were manually checked multiple times.

Glacier area errors exhibit an inverse relationship with the length of glacier margins and strongly correlate with glacier size. Numerous researchers have argued that the glacier buffer method is suitable for estimating glacial area errors because it considers the perimeter of the glaciers.^15^^,^^32^^,^^33^ This buffer method is employed to assess glacial area uncertainties in various regions, including western Canada,^33^ the Caucasus Mountains,^35^ and the TS region.^15^^,^^33^ The maximum buffer size for error estimation was set to half the anticipated shift due to misregistration, assuming only one side of the glacier would be affected. This shift could lead to a cutoff at the glacier boundaries. The error for each glacier was calculated by incorporating the glacier’s perimeter and accounting for area uncertainty. Furthermore, the buffer method was utilized to extract alpine lakes.

The uncertainty of glacier areas was calculated as follows:

(Equation 1) $$S_{\max}=L\times A$$

where S_max_ is the glacial area error (km^2^), L is the perimeter of the glacier outline (km) for each glacier, and A is the maximum error in the extraction of each glacial area (km). Landsat TM/ETM+/OLI images have a resolution of 30 m, whereas the WorldView-2 imagery provides high resolution during glacier area extraction. The maximum error considered for glacier and glacial lake extraction was set to half the pixel size of the imagery, with Landsat TM/ETM+/OLI images having a terrestrial error of 15 m and WorldView-2 images 0.25 m. Glacier area uncertainties were systematically quantified for different regions (East, North, Central TS) and time periods (1990, 2000, 2015), accounting for glacier size and perimeter length. Smaller glaciers, particularly in East TS, exhibited higher relative uncertainties due to their shorter perimeters, while larger glaciers in Central TS showed more stable estimates. The uncertainties in glacier area extraction for the East TS, North TS, and Central TS were 8.31%, 7.88%, and 5.14%, respectively, in 1990; 8.95%, 7.65%, and 5.27% in 2000; and 9.31%, 8.56%, and 5.27% in 2015. These uncertainties were further considered in estimating retreat rates, lake area changes, and runoff responses, ensuring that the conclusions regarding hydrological changes remain robust despite inherent measurement errors.

The uncertainty in lake area is primarily influenced by the spatial resolution of the imagery and potential co-registration errors. Given that the Landsat TM/ETM+/OLI sequences used in this study underwent standard terrain correction (Level 1T) and lake comparisons were conducted on an entity-by-entity basis rather than pixel-by-pixel, we assumed that co-registration errors did not significantly impact the measurements. The buffer method was employed to estimate the uncertainty in glacial lake boundaries. The pixel size represents a ground resolution of 15 m for Landsat TM/ETM+/OLI imagery and 0.25 m for WorldView-2 images. Overall, the maximum uncertainties were 14.38%, 14.99%, 15.03%, and 15.89% in 1990, 2000, 2010, and 2015, respectively.

### Quantification and statistical analysis

All statistical analyses were performed using MATLAB R2025a (MathWorks), ArcGIS 10.8.2 (Esri), and Python 3.12.3. ArcGIS was used for spatial processing, including resampling, reprojection, basin delineation, spatial aggregation, and uncertainty estimation of glacier and lake extents. MATLAB and Python were used for quantitative calculations and statistical analyses of terrestrial water storage (TWS), soil moisture (SM), snow water equivalent (SWE), climate variables, glacier and lake dynamics, and river runoff. Temporal trends were evaluated using the non-parametric Mann–Kendall test, and trend magnitudes were quantified using Sen’s slope estimator. Statistical significance was defined at *p* < 0.05 unless otherwise stated, with exact values reported in the results and figure legends. Linear regression analysis was applied to examine relationships between glacier change, meltwater contribution, and runoff dynamics, with regression coefficients and R^2^ values reported. Uncertainty in glacier and lake area extraction was estimated using a buffer-based approach to account for positional and classification errors. All statistical details, including sample sizes, significance tests, and uncertainty estimates, are provided in the results, figure legends, and supplemental information.

### Additional resources

Publicly available datasets can be accessed via the link: https://zenodo.org/records/18035651. Original code is available from the corresponding author upon reasonable request.

## References

[1] Drenkhan F., Buytaert W., Mackay J.D., Barrand N.E., Hannah D.M., Huggel C. (2022). Looking beyond glaciers to understand mountain water security. Nat. Sustain..

[2] Caro A., Condom T., Rabatel A., Champollion N., García N., Saavedra F. (2024). Hydrological response of Andean catchments to recent glacier mass loss. Cryosphere.

[3] Zhao H., Su B., Lei H., Zhang T., Xiao C. (2023). A new projection for glacier mass and runoff changes over High Mountain Asia. Sci. Bull..

[4] Li D., Lu X., Walling D.E., Zhang T., Steiner J.F., Wasson R.J., Harrison S., Nepal S., Nie Y., Immerzeel W.W. (2022). High Mountain Asia hydropower systems threatened by climate-driven landscape instability. Nat. Geosci..

[5] Pritchard H.D. (2019). Asia's shrinking glaciers protect large populations from drought stress. Nature.

[6] Xu M., Kang S., Wu H., Yuan X. (2018). Detection of spatio-temporal variability of air temperature and precipitation based on long-term meteorological station observations over Tianshan Mountains, Central Asia. Atmos. Res..

[7] Li Y.J., Ding Y.J., Shangguan D.H., Wang R.J. (2019). Regional differences in global glacier retreat from 1980 to 2015. Adv. Clim. Change Res..

[8] Li D., Lu X., Overeem I., Walling D.E., Syvitski J., Kettner A.J., Bookhagen B., Zhou Y., Zhang T. (2021). Exceptional increases in fluvial sediment fluxes in a warmer and wetter high mountain Asia. Science.

[9] Miles E., McCarthy M., Dehecq A., Kneib M., Fugger S., Pellicciotti F. (2021). Health and sustainability of glaciers in High Mountain Asia. Nat. Commun..

[10] Zhu Y., Liu S., Wei J., Wu K., Bolch T., Xu J., Guo W., Jiang Z., Xie F., Yi Y. (2025). Glacier-level and gridded mass change in river sources in the eastern Tibetan Plateau region (ETPR) from the 1970s to 2000. Earth Syst. Sci. Data.

[11] Yi Y., Zhu Y., Liu S.Y., Saifullah M., Wu K.P., Liu Q., Wei J.Y. (2025). Weakening trends of glacier and snowmelt-induced floods in the Upper Yarkant River Basin, Karakoram during 1961–2022. Adv. Clim. Chang. Res..

[12] Bolch T. (2017). Hydrology: Asian glaciers are a reliable water source. Nature.

[13] Chen Y., Zhang X., Fang G., Li Z., Wang F., Qin J., Sun F. (2020). Potential risks and challenges of climate change in the arid region of northwestern China. Reg. Sustainability.

[14] de Kok R.J., Kraaijenbrink P.D.A., Tuinenburg O.A., Bonekamp P.N.J., Immerzeel W.W. (2020). Towards understanding the pattern of glacier mass balances in High Mountain Asia using regional climatic modelling. Cryosphere.

[15] Zhang Q., Chen Y., Li Z., Xiang Y., Li Y., Sun C. (2022). Recent Changes in Glaciers in the Northern Tien Shan, Central Asia. Remote Sens..

[16] Chen Y., Li W., Deng H., Fang G., Li Z. (2016). Changes in Central Asia's Water Tower: Past, Present and Future. Sci. Rep..

[17] Farinotti D., Longuevergne L., Moholdt G., Duethmann D., Mölg T., Bolch T., Vorogushyn S., Güntner A. (2015). Substantial glacier mass loss in the Tien Shan over the past 50 years. Nat. Geosci..

[18] Liu J., Lawson D.E., Hawley R.L., Chipman J., Tracy B., Shi X., Chen Y. (2020). Estimating the longevity of glaciers in the Xinjiang region of the Tian Shan through observations of glacier area change since the Little Ice Age using high-resolution imagery. J. Glaciol..

[19] Petrakov D., Shpuntova A., Aleinikov A., Kääb A., Kutuzov S., Lavrentiev I., Stoffel M., Tutubalina O., Usubaliev R. (2016). Accelerated glacier shrinkage in the Ak-Shyirak massif, Inner Tien Shan, during 2003–2013. Sci. Total Environ..

[20] Zhang Q., Chen Y., Li Z., Fang G., Xiang Y., Li Y. (2022). Controls on Alpine Lake Dynamics, Tien Shan, Central Asia. Remote Sens..

[21] Kaldybayev A., Chen Y., Vilesov E. (2016). Glacier Change in the Karatal River Basin, Zhetysu (Dzhungar) Alatau, Kazakhstan. Ann. Glaciol..

[22] Li Y., Chen Y., Li Z. (2020). Climate and topographic controls on snow phenology dynamics in the Tienshan Mountains, Central Asia. Atmos. Res..

[23] Chen Y., Li Z., Fang G., Li W. (2018). Large Hydrological Processes Changes in the Transboundary Rivers of Central Asia. JGR Atmos..

[24] Hugonnet R., McNabb R., Berthier E., Menounos B., Nuth C., Girod L., Farinotti D., Huss M., Dussaillant I., Brun F., Kääb A. (2021). Accelerated global glacier mass loss in the early twenty-first century. Nature.

[25] Zhang G., Bolch T., Yao T., Rounce D.R., Chen W., Veh G., King O., Allen S.K., Wang M., Wang W. (2023). Underestimated mass loss from lake-terminating glaciers in the greater Himalaya. Nat. Geosci..

[26] Yao J., Chen Y., Guan X., Zhao Y., Chen J., Mao W. (2022). Recent climate and hydrological changes in a mountain-basin system in Xinjiang, China. Earth Sci. Rev..

[27] Chen H., Chen Y., Li W., Li Z. (2019). Quantifying the contributions of snow/glacier meltwater to river runoff in the Tianshan Mountains, Central Asia. Glob. Planet. Change.

[28] Shen Y.J., Shen Y., Fink M., Kralisch S., Chen Y., Brenning A. (2018). Trends and variability in streamflow and snowmelt runoff timing in the southern Tianshan Mountains. J. Hydrol..

[29] Kraaijenbrink P.D.A., Stigter E.E., Yao T., Immerzeel W.W. (2021). Climate change decisive for Asia’s snow meltwater supply. Nat. Clim. Chang..

[30] Zheng G., Bao A., Allen S., Antonio Ballesteros-Cánovas J., Yuan Y., Jiapaer G., Stoffel M. (2021). Numerous unreported glacial lake outburst floods in the Third Pole revealed by high-resolution satellite data and geomorphological evidence. Sci. Bull..

[31] Zhang G.Q., Bolch T., Allen S., Linsbauer A., Chen W.F., Wang W.C. (2019). Glacial lake evolution and glacier–lake interactions in the Poiqu River basin, central Himalaya, 1964–2017. J. Glaciol..

[32] Pfeffer W.T., Arendt A.A., Bliss A., Bolch T., Cogley J.G., Gardner A.S., Hagen J.O., Hock R., Kaser G., Kienholz C. (2014). The Randolph Glacier Inventory: a globally complete inventory of glaciers. J. Glaciol..

[33] Shangguan D.H., Bolch T., Ding Y.J., Kröhnert M., Pieczonka T., Wetzel H.U., Liu S.Y. (2015). Mass changes of Southern and Northern Inylchek Glacier, Central Tian Shan, Kyrgyzstan, during ∼ 1975 and 2007 derived from remote sensing data. Cryosphere.

[34] Tielidze L.G., Wheate R.D. (2018). The Greater Caucasus Glacier Inventory (Russia, Georgia and Azerbaijan). Cryosphere.

[35] Mukherjee K., Bolch T., Goerlich F., Kutuzov S., Osmonov A., Pieczonka T., Shesterova I. (2017). Surge-type glaciers in the Tien Shan (Central Asia). Arct. Antarct. Alp. Res..

[36] Pieczonka T., Bolch T., Junfeng W., Shiyin L. (2013). Heterogeneous mass loss of glaciers in the Aksu-Tarim Catchment (Central Tien Shan) revealed by 1976 KH-9 Hexagon and 2009 SPOT-5 stereo imagery. Remote Sens. Environ..

[37] Wang X., Guo X., Yang C., Liu Q., Wei J., Zhang Y., Liu S., Zhang Y., Jiang Z., Tang Z. (2020). Glacial lake inventory of high-mountain Asia in 1990 and 2018 derived from Landsat images. Earth Syst. Sci. Data.

[38] Deng H., Chen Y., Li Y. (2019). Glacier and snow variations and their impacts on regional water resources in mountains. J. Geogr. Sci..

[39] Yang Z., Bai P., Tian Y., Liu X. (2025). Glacier coverage dominates the response of runoff and its components to climate change in the Tianshan mountains. Water Resour. Res..

[40] Chen Y.N., Li Z., Fang G.H., Deng H.J. (2017). Impact of climate change on water resources in the Tianshan Mountians, Central Asia. Acta Geogr. Sin..

[41] Ragettli S., Immerzeel W.W., Pellicciotti F. (2016). Contrasting climate change impact on river flows from high-altitude catchments in the Himalayan and Andes Mountains. Proc. Natl. Acad. Sci. USA.

[42] Zhao Q., Zhang S., Ding Y.J., Wang J., Han H., Xu J., Zhao C., Guo W., Shangguan D. (2015). Modeling hydrologic response to climate change and shrinking glaciers in the highly glacierized kunma like river catchment, Central tian shan. J. Hydrometeorol..

[43] IPCC (2013).

[44] Li Z., Chen Y., Li Y., Wang Y. (2020). Declining snowfall fraction in the alpine regions, Central Asia. Sci. Rep..

