# Supplementary material for: Glacier change threatens Central Asia’s water towers
Source: iScience. 2026 Jan 17;29(2):114727. doi: 10.1016/j.isci.2026.114727 (PMC12887379; doi:10.1016/j.isci.2026.114727)
Supplement: Document S1. Figure S1, Table S1, and Methods S1 [file mmc1.pdf]

iScience, Volume *29*

Supplemental information

Glacier change threatens Central Asia's water towers

Qifei Zhang, Yaning Chen, Zhi Li, Gonghuan Fang, Yanyun Xiang, and Congjian Sun

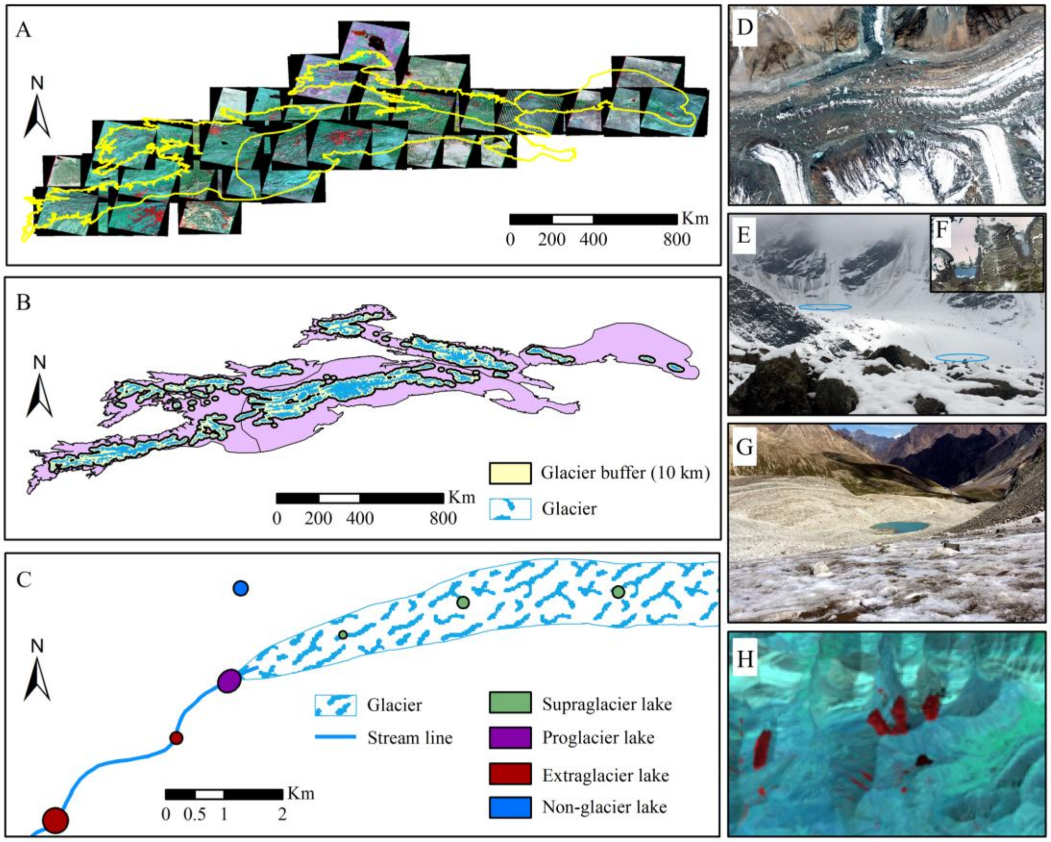

Figure S1. Flowchart of alpine lake classification in the Tien Shan

(A) Available Landsat images utilized in the Tien Shan in 2015.

(B) Extraction of alpine lakes located within a 10 km buffer zone of glaciers.

(C) Locations of various types of alpine lakes relative to glaciers.

(D, E, F, G and H) Imagery and photographs of supraglacial, proglacial, extraglacial, and non-glacial lakes in the Tien Shan.

Table S1. Variations in alpine lake number and area in Tien Shan from 1990 to 2015

Periods	East Tien Shan		North Tien Shan		Central Tien Shan		West Tien Shan	
	Number /	Area km^2	Number /	Area km^2	Number /	Area km^2	Number /	Area km^2
1990	57	2.73	460	19.79	567	48.20	625	32.31
2000	77	3.51	516	22.39	613	48.57	779	35.94
2010	78	3.70	586	24.91	725	55.22	815	38.22
2015	98	3.95	675	27.55	813	58.47	835	39.79
1990–2015	41	1.22	215	7.76	246	10.27	210	7.48
1990–2015 (%/year)	2.88	1.80	1.87	1.57	1.74	0.85	1.34	0.93

Methods S1: Supplementary methods for glacier extraction, volume estimation, area change calculation, lake extraction, and associated error assessment

At present, there are many different image classification methods for glacier mapping that we evaluated, including band ratio thresholding, supervised and unsupervised classification, the normalized difference snow index (NDSI), the decision tree classifier, the object-oriented image interpretation.[1-4] However, factors such as snow, shadowing, moraines, and water complicate data collection at glacier sites, making it challenging to ensure the accuracy of extracted information. Therefore, we employed the band ratio threshold method, an efficient and time-effective approach for distinguishing glaciers from clouds and shadows.[5] Subsequently, we manually reviewed the multispectral images using a color combination that highlights the differences between glacier (snow, ice, and debris cover) and non-glacier areas.

Glacier outlines were delineated from Landsat images for the years 1990, 2000, and 2015. We first employed a well-established semi-automated approach, utilizing the TM3/TM5 or TM4/TM6 band ratio to classify glacier and non-glacier areas. Although the spectral band-ratio method is recognized as the most effective technique for mapping debris-free glaciers,[2] this semi-automated method is commonly used in global glacier inventories.[6,7] Paul et al. [5] reported a strong agreement for clean ice, which exhibited sufficient contrast to the surrounding terrain (approximately 5% difference). However, this method is not suitable for debris-covered glaciers.[8] In such cases, visual interpretation was applied, relying on features such as terminal moraines, glacier meltwater stream heads, glacial lakes, and lateral moraines. Misclassified areas, including snow patches, cast shadows, and lakes, were manually corrected using multispectral band combinations (TM/ETM+ bands 3, 5, and 7; OLI bands 4, 6, and 7) from the Landsat imagery.

As mentioned above, uncertainties in glacier mapping are an ongoing issue. For instance, frozen water bodies, clouds, snowfields, shadows and debris cover are unavoidable factors[2,9,10] affecting the accuracy of glacier outline maps. In the Aksu River Basin, the refreezing of water bodies did not occur during the ablation periods, so this factor is neglected. To reduce uncertainty in analyzing the Landsat images, we obtained images in which there only few clouds in the 1990, 2000 and 2015 imagery. Additionally, misclassified areas such as lakes, shadows, seasonal snow cover and debris cover were manually edited out Supraglacial debris cover is a factor reducing the accuracy of the glacier outline, and about 5% of the glacier surfaces in our study area are debris-covered.[11,12] In order to identify areas with a supraglacial debris cover, we also examined high-resolution images in Google Earth, as well as the CGI and RGI inventories. To fill in minor gaps caused by poor-quality images, we used multi-temporal images from 1-3 years outside the year of classification to identify glacier margins in those gaps in coverage. Finally, all the debris-covers glacier outlines were manually checked multiple times.

The uncertainty associated with mapped glacier outlines is a critical factor in defining errors in glacier extent assessments, particularly when using satellite imagery. Image resolution significantly impacts the quality of glacier delineation[13] and, consequently, the determination of glacier-covered areas. Additionally, factors such as snow accumulation, ice lakes, and the delineation of glacial till coverage can substantially affect the accuracy of remote sensing glacier inventories.

During the extraction of glaciers, Google Earth provides a crucial reference data source, integrating 80% of the world's SRTM data and overlaying various remote sensing images. The 3D manipulation tools allow users to transform the images from multiple perspectives, providing three-dimensional surface representations that closely resemble real-world scenes. Some images in Western China, sourced from Quick Bird satellite, even reach sub-meter resolution, allowing clear differentiation between glacial and non-glacial areas, as well as glacial till distribution areas. High-resolution imagery from Google Earth is used to correct glacier boundary extraction, minimizing uncertainties.

This study also incorporated high-resolution WorldView-2 imagery (~0.5 m resolution), displayed directly on ArcMap through online topographic maps, with adjusted contrast and brightness to enhance glacial features. By superimposing manually revised glacier boundaries on these high-resolution images, we can identify erroneous areas clearly. Moreover, through repeated training, catalogers significantly improve their skills and experience in identifying glacier boundaries under various satellite image conditions and surface features, thereby enhancing extraction accuracy and accelerating cataloging progress.

To simulate topographic distribution checks of glacier boundaries, we addressed the limitations of ArcMap in simultaneously referencing remote sensing images and topographic data, achieving effective 3D visualization. This method also helps differentiate between snow and glacial till coverage based on the glacier's topography and geomorphology. Additionally, this study referenced the Randolph Glacier Inventory (RGI6.0) for extracting glacier information within the study area, loading multiple periods of glacier interpretation boundaries. Each period's glacier extraction information for the Tian Shan region was verified, with anomalies checked individually and cross-referenced against current imagery.

The estimation of glacier ice reserves in this study relied on the method of glacier area and volume scaling presented in the second glacier catalog:

$$V = A \times S^{Y} \qquad \text{(Equation S1)}$$

where V represents glacial ice reserves (km^3), S denotes the glacial area (km^2), Y is the scaling coefficient set to 1.375, and A is a constant value of 0.0365.[14]

Changes in the area of glaciers over time may reflect change or variability in climate at local and regional scales. Some limitations exist when using different time ranges and image resolutions. Despite these limitations, area delineations provide an important basis for assessing changes in glaciers at large temporal and spatial scales.

We used the annual percentage of area change (*APAC*) to reveal the rate of glacial recession. The area change for a time period is:

$$APAC = \frac{\Delta S}{S_0 \Delta \mathrm{t}} \qquad \text{(Equation S2)}$$

where ΔS is the variation of glacier area (km^2), S_0 is the glacier area at the initial status (km^2), and Δt is the time-span for period (yr).

The uncertainty associated with errors in extracting glacier and mountain lake information from various remote sensing data primarily depends on the spatial resolution of the imagery and registration errors.[15] Since the Landsat TM/ETM+/OLI images used in this study have all undergone standard terrain correction (Level 1T), and our analysis compares entire areas rather than conducting a pixel-by-pixel assessment, we conclude that co-registration errors do not significantly impact the measurement of glacier and lake areas.[16,17] The spatial resolution of Landsat TM is 30 m, consistent with most bands of the Landsat ETM+/OLI images (the panchromatic band has a higher resolution of 15 m). In contrast, WorldView-2 imagery offers higher resolution, resulting in reduced errors during the extraction of glacier and lake areas. The automatically extracted mountain lake areas exhibit a grid with more cells and rougher boundaries, but this uncertainty is further minimized through visual identification and manual editing.

Additionally, this study employs interactive post-classification comparison and editing to address identification errors. To minimize uncertainty in extracting glaciers and mountain lakes, we prioritize selecting high-quality Landsat images from optimal summer years. However, extracting information on glaciers and glacial lakes from the same month within a 1- to 3-year span is not feasible. Building on recent studies,[16,18] we use the buffer zone method to estimate extraction uncertainty for glaciers and mountain lakes. This uncertainty is derived from the concurrent uncertainty in the edge lengths of glaciers and lakes.

The maximum error considered is half a pixel of the extraction image, equating to 15 m for Landsat TM/ETM+/OLI images and approximately 0.25 m for WorldView-2 images. Using the glacier boundary buffer zone method, the uncertainty ranges for glacier area extraction across various regions of the Tien Shan from 1990 to 2015 are as follows: ~9.31% for the Eastern Tien Shan, ~8.29% for the Northern Tien Shan, and ~5.27% for the Central Tien Shan.

In extracting lakes in the Tien Shan region, this study employs the method proposed by Hanshaw and Bookhagen (2014)[19] to assess the uncertainty associated with lake area extraction. We assume that the uncertainty in the lake boundaries after manual correction follows a Gaussian distribution. As pixels representing lake water are usually surrounded by mixed pixels, the error in lake boundaries is ±0.5 pixels, reflecting the spatial resolution of Landsat imagery. This value is then multiplied by half the area of a single pixel, under the assumption that the uncertainty for each pixel corresponds to half a pixel, as expressed in the following formula:

$$E_i = \frac{p}{g} \times \frac{g^2}{2} \times 0.6872 \quad \text{(Equation S3)}$$

$$ERR = \frac{\sum_i^n E_i}{A} \times 100\% \quad \text{(Equation S4)}$$

Where *p* represents the perimeter of the mountain lake (m); *g* is the spatial resolution of the Landsat satellite image (m); 0.6872 is the correction coefficient (approximately 69% of the edge pixels are subject to error); ERR is the relative error of the total lake area (%); E_i is the area of lake i (km^2); A is the total area of all mountain lakes (km^2).

The pixel size represents a ground resolution of 15 meters for the Landsat TM/ETM+/OLI imagery and 0.25 meters for the WorldView-2 images. Overall, the maximum uncertainties were 14.38%, 14.99 %, 15.03 %, and 15.89% in 1990, 2000, 2010, and 2015, respectively. The repeated mapping results of the glacial lake samples in the East Tien Shan indicate that the error in the total area of alpine lakes in the year 2000 was 5.64%, and in 2015 it was 7.6%. Concurrently, the assessment of lake areas less than 0.1 km^2 shows that the lake area errors for the years 2000 and 2015 were 12.47% and 10.86%, respectively.

Various types of lakes have formed in the Tien Shan, including non-closed lakes, which complicate monitoring the continuous replenishment from glacier meltwater and upstream precipitation. This can lead to significant errors in results. To accurately analyze the factors driving glacial lake expansion in the Tien Shan and to monitor the main influences on this expansion, this study integrated high-resolution WorldView-2 imagery, Google Earth data, and Landsat TM/ETM+/OLI imagery, along with 30 m resolution DEM data, to identify and classify lakes in the Eastern Tien Shan. Additionally, to better assess the impact of climate variability on lake changes, water bodies affected by human activity, such as reservoirs and ditches, were excluded from the analysis.

SUPPLEMENTAL REFERENCES

1. Bolch, T. (2007). Climate change and glacier retreat in northern Tien Shan (Kazakhstan/Kyrgyzstan) using remote sensing data. Global Planet. Change *56*, 1–12. http://doi.org/https://doi.org/10.1016/j.gloplacha.2006.07.009.
2. Kaldybayev, A., Chen, Y.N., and Vilesov, E. (2016). Glacier change in the Karatal river basin, Zhetysu (Dzhungar) Alatau, Kazakhstan. Ann. Glaciol. *57*, 11–19. http://doi.org/10.3189/2016AoG71A005.
3. Racoviteanu, A., and Williams, M.W. (2012). Decision Tree and Texture Analysis for Mapping Debris-Covered Glaciers in the Kangchenjunga Area, Eastern Himalaya. Remote Sens. *4*, 3078–3109. http://doi.org/10.3390/rs4103078.
4. Willmes, S., Bareiss, J., Haas, C., and Nicolaus, M. (2009). Observing snowmelt dynamics on fast ice in Kongsfjorden, Svalbard, with NOAA/AVHRR data and field measurements. Polar Res. *28*, 203–213, 10-1111.
5. Paul, F., Barrand, N.E., Baumann, S., Berthier, E., Bolch, T., Casey, K., Frey, H., Joshi, S.P., Konovalov, V., and Bris, R.L. (2013). On the accuracy of glacier outlines derived from remote-sensing data. Ann. Glaciol. *54*, 171–182.
6. Aizen, V.B., Kuzmichenok, V.A., Surazakov, A.B., and Aizen, E.M. (2006). Glacier changes in the central and northern Tien Shan during the last 140 years based on surface and remote-sensing data. Ann. Glaciol. *43*, 202–213. http://doi.org/10.3189/172756406781812465.
7. Pan, B.T., Zhang, G.L., Wang, J., Cao, B., Geng, H.P., Zhang, C., and Ji, Y.P. (2012). Glacier changes from 1966–2009 in the Gongga Mountains, on the south-eastern margin of the Qinghai-Tibetan Plateau and their climatic forcing. The Cryosphere *6*, 1087–1101. http://doi.org/10.5194/tc-6-1087-2012.
8. Paul, F., and Linsbauer, A. (2012). Modeling of glacier bed topography from glacier outlines, central branch lines, and a DEM. Int. J. Geogr. Inf. Sci. *26*, 1173–1190.
9. Barry, R.G. (2006). The status of research on glaciers and global glacier recession: a review . *Prog. Phys. Geog. 30*, 285–306.
10. Gardner, A.S., Moholdt, G., Cogley, J.G., Wouters, B., Arendt, A.A., Wahr, J., Berthier, E., Hock, R., Pfeffer, W.T., Kaser, G. et al. (2013). A Reconciled Estimate of Glacier Contributions to Sea Level Rise: 2003 to 2009. *Science*. *340*, 852-857, 10–1126.

11. Duethmann, D., Bolch, T., Farinotti, D., Kriegel, D., Vorogushyn, S., Merz, B., Pieczonka, T., Jiang, T., Su, B. D. and G Untner, A. (2015). Attribution of Streamflow Trends in Snow and Glacier Melt-Dominated Catchments of the Tarim River, Central Asia. *Water Resour. Res. 51*, 4727–4750.
12. Pieczonka, T. and Bolch, T. (2015). Region-Wide Glacier Mass Budgets and Area Changes for the Central Tien Shan Between ~1975 and 1999 Using Hexagon KH-9 Imagery. *Global Planet. Change*. 128, 1–13.
13. Hagg, W., Mayer, C., Lambrecht, A., Kriegel, D. and Azizov, E.(2013). Glacier Changes in the Big Naryn Basin, Central Tian Shan. *Global Planet. Change*. *110*, 40–50.
14. Grinsted, A., 2013. An estimate of global glacier volume. The Cryosphere Discussions 7, 141–*151*, 10-5194. http://doi.org/10.5194/tc-7-141-2013.
15. Hall, D.K., Bayr, K.J., Schöner, W., Bindschadler, R.A., and Chien, J.Y.L. (2003). Consideration of the errors inherent in mapping historical glacier positions in Austria from the ground and space (1893–2001). Remote Sens. Environ. *86*, 566–577. http://doi.org/10.1016/S0034-4257(03)00134-2.
16. Petrov, M.A., Sabitov, T.Y., Tomashevskaya, I.G., Glazirin, G.E., Chernomorets, S.S., Savernyuk, E.A., Tutubalina, O.V., Petrakov, D.A., Sokolov, L.S., and Dokukin, M.D. (2017). Glacial lake inventory and lake outburst potential in Uzbekistan. Sci. Total Environ. *592*, 228–242.
17. Gardelle, J., Arnaud, Y., and Berthier, E. (2011). Contrasted evolution of glacial lakes along the Hindu Kush Himalaya mountain range between 1990 and 2009. Global Planet. Change *75*, 47–55. http://doi.org/10.1016/j.gloplacha.2010.10.003.
18. Zheng, G.X., Bao, A.M., Li, J.L., Zhang, G.Q., Xie, H.J., Guo, H., Jiang, L.L., Chen, T., Chang, C., and Chen, W.F. (2019). Sustained growth of high mountain lakes in the headwaters of the Syr Darya River, Central Asia. Global Planet. Change *176*, 84–99. http://doi.org/https://doi.org/10.1016/j.gloplacha.2019.03.004.
19. Hanshaw, M.N., and Bookhagen, B. (2014). Glacial areas, lake areas, and snow lines from 1975 to 2012: status of the Cordillera Vilcanota, including the Quelccaya Ice Cap, northern central Andes, Peru. The cryosphere *8*, 359–376. http://doi.org/10.5194/tc-8-359-2014.
